# Using a SMALP platform to determine a sub-nm single particle cryo-EM membrane protein structure

**DOI:** 10.1016/j.bbamem.2017.10.005

**Published:** 2018-02

**Authors:** Mayuriben Parmar, Shaun Rawson, Charlotte A. Scarff, Adrian Goldman, Timothy R. Dafforn, Stephen P. Muench, Vincent L.G. Postis

**Affiliations:** a Biomedicine Research Group, Faculty of Health and Social Sciences, Leeds Beckett University, LS1 3HE, UK; b School of Biomedical Sciences, Faculty of Biological Sciences, University of Leeds, LS2 9JT, UK; c School of Molecular and Cellular Biology, Faculty of Biological Sciences, University of Leeds, LS2 9JT, UK; d Astbury Centre for Structural Molecular Biology, University of Leeds, LS2 9JT, UK; e Department of Biosciences, Division of Biochemistry, University of Helsinki, Helsinki, Finland; f School of Biosciences, University of Birmingham, Edgbaston, Birmingham, B15 2TT, UK

**Keywords:** SMALP, Electron microscopy, Membrane proteins, AcrB

## Abstract

The field of membrane protein structural biology has been revolutionized over the last few years with a number of high profile structures being solved using cryo-EM including Piezo, Ryanodine receptor, TRPV1 and the Glutamate receptor. Further developments in the EM field hold the promise of even greater progress in terms of greater resolution, which for membrane proteins is still typically within the 4–7 Å range. One advantage of a cryo-EM approach is the ability to study membrane proteins in more “native” like environments for example proteoliposomes, amphipols and nanodiscs. Recently, styrene maleic acid co-polymers (SMA) have been used to extract membrane proteins surrounded by native lipids (SMALPs) maintaining a more natural environment. We report here the structure of the *Escherichia coli* multidrug efflux transporter AcrB in a SMALP scaffold to sub-nm resolution, with the resulting map being consistent with high resolution crystal structures and other EM derived maps. However, both the C-terminal helix (TM12) and TM7 are poorly defined in the map. These helices are at the exterior of the helical bundle and form the greater interaction with the native lipids and SMA polymer and may represent a more dynamic region of the protein. This work shows the promise of using an SMA approach for single particle cryo-EM studies to provide sub-nm structures.

## Highlights

• The first sub nm single particle reconstruction of a membrane protein using a SMALP scaffold
• The ability to maintain a native like environment for structural studies holds great promise.
• The resulting map is consistent with high resolution crystal structures and other EM derived maps.

## Graphical abstract

**Figure:**
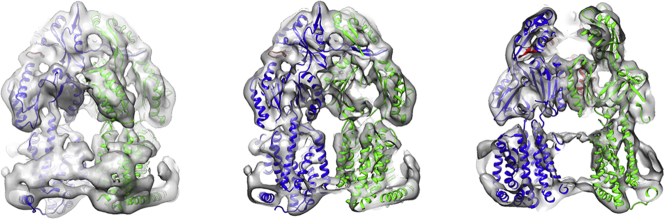

## 1 Introduction

Despite the biological importance of membrane proteins and their relevance for drug targeting, our structural understanding of this class of proteins is much poorer than that of their soluble counterparts. A range of factors contribute to this, including difficulties in protein expression, which may be brought about through cellular toxicity, membrane crowding and protein mis-folding [1]. Moreover, the requirement to stabilise the protein outside of the membrane environment, usually with detergents, often results in lower yields than their soluble counterparts. The presence of detergent can also hinder crystal growth, with the resulting micelle limiting crystal contacts, a major hurdle for membrane protein crystallography [2].

A significant change in the landscape of membrane protein structural biology has been brought about by the advances in cryo-electron microscopy (cryo-EM) [3], [4]. A cryo-EM approach presents a number of key advantages to studying membrane proteins, for example although the protein concentrations required are often ~ 1 mg/ml only 3–5 μl is required per grid resulting in only μg levels of protein being used rather than the mg quantities typically required for X-ray crystallography and NMR studies. Other significant benefits are the absence of the requirement for highly-ordered crystals (X-ray crystallography) or long data collection times on non-frozen specimens (NMR). Moreover, EM is a powerful approach to capturing different conformational states of a protein or protein complex, exemplified by the ribosome and Slo2.2 channel [5], [6]. This can provide a deeper understanding into the different conformational states of these membrane proteins.

With the drive towards higher resolutions and the ability to capture conformational states, comes a very important requirement for all membrane protein studies: keeping the target in a native-like environment. To this end several systems are available with the most commonly used being detergents. Their amphipathic nature aims to mimic the membrane environment and stabilise the protein in aqueous solutions [7]. Detergent micelles are only a rough approximation of the natural membrane environment and they tend to remove the native lipidic components surrounding the protein which often leads to the modification of the protein function [8], [9], [10], [11]. Furthermore, the identification of the optimal detergent(s) and buffer conditions for protein stability is often challenging [12], [13]. Recently, the use of amphipathic polymers (amphipols) for stabilizing membrane proteins has become more common. Noticeable example of cryo-EM studies in amphipol are transient receptor potential (TRP) channels and the ryanodine receptor [14], [15]. In nanodiscs, amphipathic protein scaffolds are used to entrap membrane proteins within a lipid bilayer. This approach has been used to great effect with TRPAV1 to provide a more “native” like environment [16]. However, both approaches still require the use of detergent in their early stages which still leads to the native lipids being stripped away. Lipids need to be reintroduced at a later stage and the exact composition to use with the selected membrane protein can be difficult to deduce [17].

A rising alternative which might address this problem is the use of styrene maleic acid copolymers (SMA) [18], [19]. An alternating cluster of hydrophobic styrene and hydrophilic maleic acid moieties, which render the polymer amphipathic allows their interaction with biological membrane and the solubilisation of membrane proteins into a structure called a styrene maleic acid lipo-particle (SMALP). This nanostructure includes a membrane protein surrounded by its native lipids encircled by polymers. The use of SMALPs has been shown to maintain the activity of proteins while conventional detergents such as DDM can show a reduced activity [20], [21], [22]. Therefore, SMALP provide a stable environment in which proteins can be studied by X-ray crystallography and a range of other biophysical approaches (CD, AUC, SAXS and SANS) [23], [24]. Moreover, the SMALP platform has already been shown to be a valid approach for negative stain EM which offers modest resolution to structure determination (> 15 Å) [22]. For the SMALP platform to become a versatile replacement to conventional approaches for membrane protein structural studies, it is essential that it is applicable to single particle cryo-EM approaches. To this end, we report the first sub-nm single particle cryo-EM reconstruction of a membrane protein, the drug efflux pump AcrB, extracted, purified and visualised in a SMALP. This shows that SMALPs offer an alternative approach to detergents, amphipol and nanodisc. With the developments in EM and the SMALP technology, this could provide a powerful approach to membrane protein structure determination in more native like environments.

## 2 Methods

### 2.1 Protein overexpression and purification

AcrB bearing a C-terminal octahistidine tag was over-expressed through auto-induction using the C43(DE3) *E. coli* strain. Following cell disruption AcrB in its native trimeric form (~ 340 kDa) was extracted from mixed inner and outer *E. coli* membranes using 2.5% (*w*/*v*) SMA which was incubated for 2 h at room temperature as described previously [22], [25]. The final buffer for the SMALP sample was 50 mM Tris-HCL pH 8.0, 500 mM NaCl. The SMA polymer is based around a styrene-to-maleic–acid ratio of 2:1 and the raw polymer was sourced from Cray Valley and the SMA synthesised as described in Lee et al. [26].

### 2.2 Negative stain electron microscopy

To assess sample quality negative stain grids were prepared and examined. Briefly, carbon-coated grids were glow-discharged for 40 s in a Pelco glow discharge unit. Following charging, 3 μl of the AcrB SMALP complex (20 μg/ml) was added to the grid and stained with uranyl acetate, as previously described [22]. All negative stain grids were imaged using a Technai T12 microscope fitted with a LaB6 filament operating at 120 kV with a nominal magnification of 30,000 × on a 2 k × 2 K Gatan CCD camera (Å/pixel value of 4.2). Micrographs were picked and reference free classes generated using EMAN 2 [27].

### 2.3 Cryo electron microscopy

Cryo-EM grids were prepared by applying 3 μl of ~ 1 mg/ml AcrB onto a glow discharged (20 s) gold quantifoil grid (2.1 mesh). To minimize the presence of divalent metal ions which destabilize the SMA polymer, grids were blotted with Ash-free Whatman filter paper (No. 50) using the Vitrobot Mark IV. Blotting was carried out for 6 s using a blot force of 6 before being plunge frozen in liquid ethane. Data was collected in-house at the Astbury Biostructure facility on a G2 Titan Krios fitted with a Falcon III direct electron detector operating in integrating mode. A total of 3400 micrographs were collected at an Å/pixel of 1.065. Micrographs were motion corrected in MotionCorr2 and CTF values determined using Gctf [28], [29]. Micrographs were manually picked in RELION to generate initial 2D references [30]. These references were then used to autopick the remaining data resulting in ~ 400,000 particles, binned by a factor of two resulting in Å/pixel of 2.13. Subsequent 2D classification and removal of particles which belonged to poorly resolved classes resulted in ~ 90,000 particles. The resulting classes showed clear and distinct classes typical of AcrB. After further rounds of 2D classification the particle number was reduced to 36,421. To further improve the particle stack and remove heterogeneity 3D classification was carried out, after which the particle number was reduced to 26,480. Initially 3D reconstructions were generated using the RELION 2.0 3D Classification procedure with a low-resolution ellipsoid as a starting model for refinement using C3 symmetry to account for the trimeric structure. To ensure there was no model bias we also carried out initial model generation using the stochastic gradient descent in RELION 2.1 with a subset of the particles (~ 9000) [31] which give a consistent result with that generated with a starting model. Following the 3D auto-refinement procedure the resulting model had a resolution of 8.8 Å, as determined by the 0.143 cut-off criteria and the final model had an automatically determined b-factor of − 907 applied. The previously solved crystal structure (PDB ID: 4ZLJ) was docked within the map using Chimera with a correlation of 0.93 at 8.8 Å. No improvement in resolution or map quality was observed in the un-binned data. The model has been deposited within the Electron Microscopy Database (EMDB) with accession number EMD-3887.

## 3 Results

### 3.1 Preparation and characterization of AcrB SMALPs

**Fig. 1:**
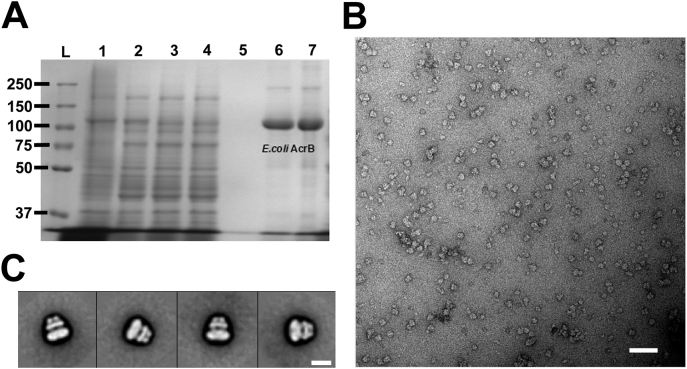
**A**) SDS PAGE gel of the purified His-tagged AcrB (~ 100 kDa as a monomer) in SMA, L = Precision plus dual ladder, 1 = total membrane protein, 2 = supernatant, 3 = flowthrough, 4 = wash 1, 5 = wash 2, 6 & 7 = elution in 300 mM imidazole. Note both of these lanes were pooled before using. **B**) Negative stain micrograph of AcrB showing high purity and monodispersity of the sample. Scale bar represents 100 Å. **C**) Representative negative stain class averages of AcrB, the scale bar in the bottom right represents 500 Å.

His-tagged AcrB was successfully overexpressed in *E. coli* and the resulting purified sample ran on SDS-PAGE showing no significant degradation products or contaminants (Fig. 1A). The samples aggregation/monodispersity was assessed by negative stain electron microscopy prior to cryo-EM grids preparation. The resulting micrographs showed good particle distribution, in agreement with previously obtained results for AcrB images (Fig. 1B). The resulting reference free classes showed typical AcrB features consistent with our previous work and that of others, on AcrB ((Fig. 1C) [22], [32]).

### 3.2 Single particle cryo-EM electron microscopy

**Fig. 2:**
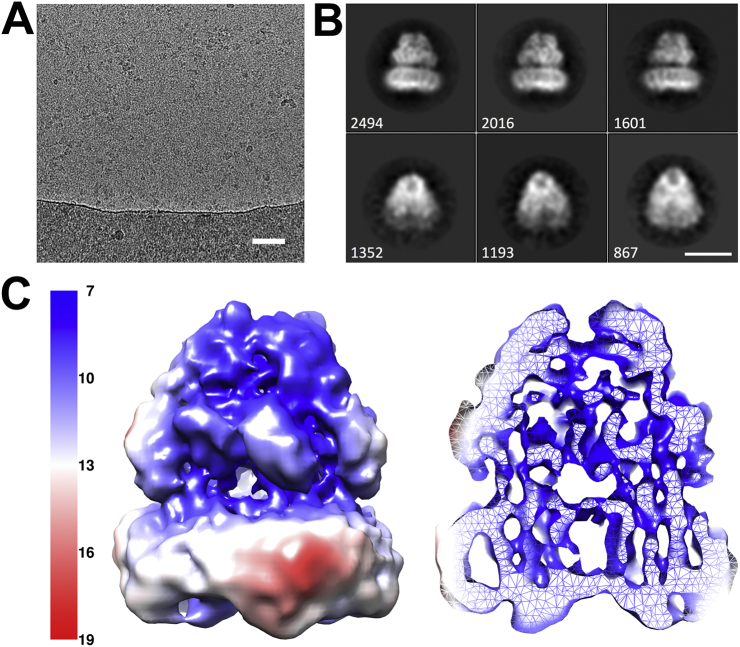
**A**) Representative micrograph of a cryo-EM AcrB grid, scale bar represents 500 Å. B) Representative 2D classes for the AcrB single particle cryo-EM dataset with side views and high angle views shown on the top and bottom row, respectively. The corresponding particle number in each class is shown on the bottom left. The white scale bar represents 100 Å. **C)** The AcrB 8.8 Å single particle reconstruction coloured by local resolution and shown as a surface (left) and slice through (right). Higher resolution is seen within the soluble “vestibule” region with the membrane region being more poorly resolved.

To investigate the suitability of the SMA polymer for single particle cryo-EM we collected a modest sized data set (3400 micrographs) of the AcrB/SMA complex (Fig. 2A). After autopicking the data and removing the poor-quality particles through iterative 2D and 3D classifications, a total of 26,480 particles remained that produced good quality classes (Fig. 2B). The resulting 3D reconstruction of AcrB had a global resolution of 8.8 Å and showed a “typical” architecture with features such as the pore and TolC docking domains that form a dome-like structure, which protrudes from the periplasmic surface of the membrane [33] (Fig. 2C). Analysis of the local resolution shows a distinct difference between the soluble and membrane bound regions with a higher resolution in the soluble domain of around 7–8 Å. Therefore, the secondary structure elements could be assigned within the vestibule region. The “base” of the AcrB/SMALP complex showed an enlargement around the protein structure about the membrane-spanning region, consistent with an SMA/phospholipid envelope surrounding the protein trimer (Fig. 2C). This region is denser than that seen for typical detergent extracted proteins, which may represent the ability of the polymer to closely pack together and form a denser “shell” than the branched detergents. The resolution within the membrane domain is lower than that of the soluble region at around 8–9 Å with helical density present. However, although helical bundles could be resolved, not all individual helices could be resolved at this resolution.

### 3.3 Comparisons to previously solved AcrB structures

The structure of AcrB has been solved previously by X-ray crystallography and EM allowing us to compare for the first time a structure derived from detergent and SMA extraction at the secondary structure level. Although high resolution structures have been obtained by X-ray crystallography, more modest structures have been reported by an EM approach [32], [34], [35], [36]. A recent study by Wang et al., reported the AcrAB-TolC apo complex to 6.5 Å resolution [34]. In this instance, the larger AcrAB-TolC complex which had been engineered to increase stability through crosslinkers, was extracted using amphipol. In both cases the soluble “vestibule” domain shows a significantly higher resolution than the membrane domain.

**Fig. 3:**
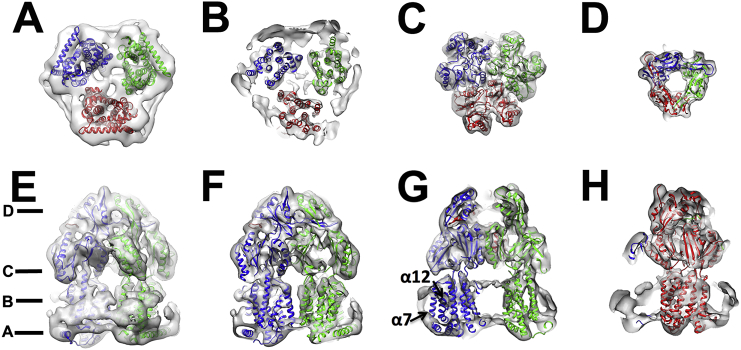
Fitting of the AcrB crystal structure within the EM derived map. **A**–**D**) A slice through from the base of AcrB to the top showing the fit of the crystal structure with each monomer coloured separately (PDB ID: 4ZLJ) within the map. The approximate position of each slice through is shown in E. **E**–**H**) A slice through from the side of AcrB. The transmembrane helices α7 and α12, form a single monomer, which are poorly resolved within the EM map are labelled in G.

Fitting of the AcrB crystal structure within our cryo-EM map shows that overall it is well accommodated with no significant difference in the two structures (Fig. 3). In the soluble domain, the individual α-helices are well defined along with the β-sheet, although at this resolution none of the β-strands can be resolved (Fig. 3 C-D). The membrane region is more poorly defined with helical bundles, rather than individual helices, being generally observed. Moreover, the C-terminal helix (TM12) is poorly defined by the map as is TM7 which is at the exterior of the helical bundle and is the helix that would interact most with the native lipids and SMA polymer (Fig. 3G). Therefore, although the SMA polymer does not result in any significant alteration to the global secondary structure in AcrB it may cause an increase of the mobility of the helices it interacts with, resulting in them being more poorly defined. The density of the polymer is also significantly stronger than that seen for detergents such as DDM and is more consistent with the density expected for a nanodisc (Fig. 3B, E).

A detailed single particle cryo-EM study on the AcrAB-TolC complex in the presence of different inhibitors provided an important leap in our understanding of the mechanical cycle of the efflux pump. For this study, the complex was initially extracted in DDM before detergent exchange into an amphipol support. Interestingly, the resolution obtained for the full complex varies depending on the conformational state and on the binding of ligands. Moreover, different parts of the complex display differing resolutions, possibly reflective of their “rigidity” within the structure [34]. The worst resolved region in all the reconstructions generated was, as with the SMA extracted AcrB structure, in the membrane bound region with the resulting reconstructions showing features consistent with the membrane spanning helices but no side chain or loop density. The vestibule region of AcrB was better defined and a small molecule inhibitor could be identified in this domain. It should also be noted that in both of these studies, the presence of the TolC aids in particle identification and alignment. Moreover, the full complex was engineered to contain a range of cross-linked sites to improve stability, which would also account for the differences seen in resolution between the isolated SMA extracted AcrB and the full complex isolated in amphipol.

### 3.4 Improvements in grid quality when accounting for divalent metals

A significant limitation with the SMA platform is the sensitivity of the polymers to divalent metal ions, resulting in destabilization of the membrane protein. Several conditions can alter the polymer solubility, one of which is the presence of divalent metal such as Ca^2 +^, Mg^2 +^, Mn^2 +^ and Zn^2 +^ [19], [26]. The standard Whatman filter paper commonly used for blotting of cryo-EM grids has a high concentration of Ca^2 +^ and other divalent metals. Upon blotting, the resulting film on the grid has a very low volume, which, when in contact with the filter paper for a prolonged time (~ 6 s), causes a significant increase in the local concentration of divalent metal ions, as noted with studies on Acto-Myosin-S1 [37]. For this reason, grids were prepared using Ash-less filter paper, which has a lower concentration of divalent metals, to minimize the potential effect of divalent metal ions destabilizing the SMA polymer. This resulted in better grids with a higher proportion of useable particles in the data processing.

## 4 Conclusions

Single particle cryo-EM has become a significant player in the membrane protein structure field and offers significant advantages over other traditional techniques [3], [4]. Not least is the ability to study membrane proteins in more “native” environments. Currently, EM studies in intact cells do not afford the same high resolution as single particle studies and so the membrane environment must be mimicked in as near to native state as possible. Detergents offer a crude approximation but can still provide high resolution structural information [38]; nanodisc and saposins are a promising approach to mimic the bilayer environment but still require detergent extraction of the source protein, removing many of the native lipids [16], [39]. The use of SMA polymers offers some distinct advantages in maintaining the native lipids and has been shown to display improved activity when compared to DDM extracted systems [22], [26]. However, although negative stain electron microscopy has been achieved with this platform, to date a sub-nm cryo-EM reconstruction has not been achieved. Here we show that the SMA polymer does permit sub-nm resolution reconstructions to be achieved through single particle cryo-EM.

In comparison to other single particle cryo-EM reconstructions conducted in DDM and amphipol, the SMA polymer is a viable alternative. An equivalent resolution to the SMA extracted AcrB was achieved for the full AcrB-TolC-MacA complex (8.2 Å) through single particle cryo-EM using an AcrBA fusion protein and the MacA-TolC hybrid protein and amphipols rather than detergent [36]. However, there is noticeable reduction in resolution when compared to the TolC-AcrA-AcrB complex, which was also in amphipol by Wang et al. [34]. There are a number of factors which play a role in this: the first is that the higher resolution full TolC-AcrA-AcrB complex was engineered to contain additional cross linkers providing a more stable complex. When comparing the resolution of the full complex to the individual AcrB structure, the better alignment accuracies for downstream processing may also play a role. A further consideration is the stability of SMA extracted membrane proteins within the air-water interface of the cryo-EM grids which impacts on the overall quality of the resulting single particles [40]. The third factor could be that in providing a more “native” environment which allows for higher activity when compared to DDM or amphipol, the SMA polymer permits a more dynamic membrane region. Studies on ompX have shown an increase in flexibility through changes in detergents [41]. Moreover, it has been shown that the lipids within a SMALP population can interchange, representing a dynamic system [42], [43]. Although for some systems this may preclude higher resolutions, this may permit a better understanding of the full conformational dynamics of a system. The flexibility maybe reduced through the addition of binding partners or inhibitors that have been shown to stabilise proteins and protein complexes. Moreover, the increased activity and the ability to conduct a range of experiments including structural studies on challenging membrane systems in the same membrane mimic makes a SMALP approach attractive for characterising membrane protein structure and function.

## Transparency document

Transparency document. Image 1

## Figures and Tables

**Supplemental Fig. 1:**
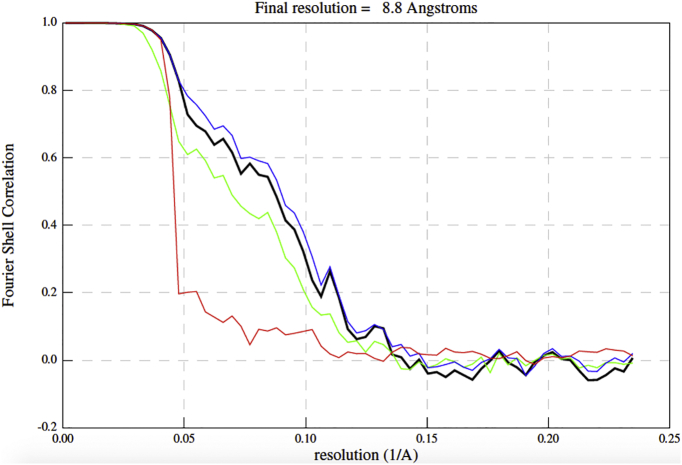
Gold Standard FSC curves for AcrB SMALP reconstruction at 8.8 Å resolution showing the Fourier shell correlation for the corrected maps (black), unmasked maps (green), masked maps (blue) and phase randomized maps (red).
